# Speculation Is Not Evidence: A Response to Conviction Is Not Commission

**DOI:** 10.1177/08862605261493525

**Published:** 2026-10-08

**Authors:** Ardavan M. Khoshnood, Kristina Sundquist

**Affiliations:** 1 Lund University, Malmö, Sweden

**Keywords:** criminology, sexual assault, cultural contexts, ethnicity, violent offenders

## Abstract

This response addresses a commentary by Orhan and Bal on our study of immigrant background and rape+ conviction in Sweden. We agree that conviction data do not capture all offending and that bias may occur at different stages of the criminal justice process. Our disagreement concerns the commentary’s movement from the possibility of such bias to an interpretation in which unequal legal treatment is treated as an explanation for the associations observed in our study. The commentary does not measure police, prosecutorial, judicial, or legal-defense bias in our cohort and therefore cannot establish that these mechanisms account for our findings. Conviction remains a legally established individual-level outcome and an accepted source in criminological research, while alternative measures of offending also have important limitations. The associations in our study were observed across several immigrant-background categories and followed a clear gradient by duration of residence in Sweden. A general reference to possible discrimination does not explain this pattern or its magnitude. We also reject the claim that discussion of prevention or possible sociocultural mechanisms is inappropriate when such interpretations are presented cautiously and as hypothesis-generating. Research on sensitive subjects requires precise language, transparent methods, and explicit recognition of limitations. It should not, however, replace observed empirical associations with unmeasured alternative explanations or avoid discussing findings because they are socially contentious.

## Introduction

As researchers, we welcome a critical discussion of our article on immigrant background and rape+ conviction in Sweden (Khoshnood et al., 2026). We therefore read the commentary by Orhan and Bal (2026) with great interest. Even though the commentary has some good points which deserve discussion, much of it rely on assumptions and speculations: that is, because bias may exist somewhere in the criminal justice process, our findings should primarily be read as evidence of unequal legal treatment rather than as a pattern in rape+ convictions. Furthermore, these speculations are not supported by the commentary itself.

The criticism can broadly be grouped into three main categories: (a) methodological concerns about conviction as an outcome variable, (b) claims about unmeasured bias and confounding, and (c) ethical concerns about language, prevention, and the discussion of sociocultural mechanisms.

Our study, just like other register-based studies, has limitations. These limitations were clearly stated and discussed in our study. With that said, we do not object to the recognition of these limitations as stated in the commentary, but to the way it moves from possible bias to high-degree explanatory certainty. The authors of the commentary do not provide evidence that potential police bias, prosecutorial bias, judicial bias, or unequal access to legal defense explains the associations observed in our study. They simply state that such mechanisms exist. That is an important distinction.

## Conviction and Inference

Conviction does not equal commission even in one of the most legally developed countries in the world, that is, Sweden. This distinction is important, but it was not hidden in our study. We explicitly studied rape+ convictions and also specified the relevant chapters and sections of the Swedish Criminal Code and the crime codes from the Swedish National Council for Crime Prevention (Brå) that were used. The outcome of the study was therefore entirely transparent.

We agree that conviction data have limitations and do not capture all offending. However, conviction is still a recognized criminal justice outcome and one of the established sources used to measure criminal behavior (Aebi & Linde, 2014; Thornberry & Krohn, 2003). The important question is thus not whether conviction captures all offending, but whether rape+ conviction is a valid operational outcome for our research question. With respect to sexual offences such as rape, conviction data may not capture all committed rapes, but they remain a legally established individual-level outcome. Other options in large-scale studies with high statistical power also have significant limitations. For example, suspect data would be more vulnerable to the raised concerns about selection, suspicion, and bias raised in the commentary; police reports are incident-based and affected by reporting and classification practices; self-reported offending is difficult to validate in serious sexual offences; and victimization surveys cannot provide the same legally established individual-level offender outcome (National Academies of Sciences, Engineering, and Medicine, 2016; Thornberry & Krohn, 2003).

The commentary seems to suggest that since bias may occur somewhere in the criminal justice process, conviction-based studies cannot support meaningful inference. That conclusion has by no means been validated by the authors of the critique against our study. If we would follow their logic, the many published studies based on convictions cannot be trusted, and almost all conviction-based research in criminology, forensic psychiatry, recidivism research and legal studies would be called into question. We agree that possible bias calls for caution, transparency, and clear limitation statements; it does not, however, make convictions to invalid research outcomes.

## The Question on Bias

A central part of the commentary is that legal and institutional bias may explain our findings. We do not reject the possibility that a certain bias may occur in the criminal justice process, and we have also mentioned this in our own article (p. 383). However, there is an important difference between stating that bias may affect conviction patterns and stating that bias explains the associations shown in our study. The commentary has not been able to measure any type of bias in our cohort. This is important since the authors of the commentary applies one standard to the potential explanations behind the findings in our study and another standard to their own alternative explanation. It deems our observed associations as insufficient because they may reflect bias. At the same time, their own interpretation, unequal conviction patterns, is presented without any valid analysis of our cohort.

The same problem is seen in the discussion on confounders. While additional variables could be relevant in future studies, our study adjusted for several important covariates. It is also important to separate confounders of importance for our study from legal-process variables. Language proficiency, legal literacy, interpretation quality, and access to legal defense may be important for understanding the criminal justice process, but they cannot be used as a substitute for offender characteristics.

The evidence from previous studies presented in the commentary is also largely indirect. Studies on general legal inequality, Dutch sentencing, Swedish court interaction, or Swedish economic crime investigations do not show that these mechanisms explain the rape+ conviction patterns in our cohort. On the contrary, a more relevant source is Brå’s report on the Swedish criminal justice system’s handling of rape cases, stated that the data did not include discrimination. Although suspects with foreign origin were prosecuted and convicted more often than suspects with Swedish origin, Brå found that the differences between the groups could be explained by differences in the nature of the cases and the strength of the evidence, while also noting that undetected differences could remain (Holmberg & Lewenhagen, 2019).

Finally, the associations shown in our results were not limited to one single subgroup; they were seen across several immigrant-background categories, with the highest odds among those born outside Sweden, those arriving at age 15 or older, and those who had lived in Sweden for a shorter period (Khoshnood et al., 2026). The residence-time gradient was also clear: among those born outside Sweden, the fully adjusted odds ratio was 2.73 for those who had lived in Sweden for more than 10 years, 4.44 for those who had lived in Sweden for 5 to 10 years, and 6.90 for those who had lived in Sweden for less than 5 years. If bias is suggested as a broad explanation related to immigrant background, it must also explain why the associations were not uniform across immigrant-background groups, but instead followed a gradient. In addition, a general reference to possible bias does not explain this magnitude, consistency, or gradient. If the commentary argues that discrimination explains this pattern which needs to be shown rather than assumed.

## Precision, Prevention, and Ethics

We agree that studies on sensitive subjects such as sexual violence and immigrant background require careful language. However, careful language does not mean avoiding sensitive findings. Bias-free language, as described by the APA, does not mean that researchers should avoid studying migration background, criminal convictions, or other socially sensitive variables when these variables are clearly defined and limitations are stated.

We also disagree with the claim that our discussion of prevention exceeds what conviction data can support. A large part of the literature on sexual offending, risk assessment, and recidivism is based on convicted or otherwise legally processed offenders (e.g., Hanson & Morton-Bourgon, 2005). Knowledge of unreported offending is impossible to obtain, and it is not needed in order to have a discussion on the question of prevention. Careful use of the best available data, together with clear statements about what the data can and cannot show, is the only available option to obtain more knowledge and improve preventive measures.

With respect to sociocultural mechanisms, our study did not claim that immigrant background is identical with culture, religion, ethnicity, language, or legal status. We explicitly described acculturation and integration as complex concepts and used proxies such as age at arrival and years lived in Sweden. We also stated that these findings should be regarded as hypothesis-generating. Discussing possible sociocultural mechanisms in sexual violence is not in itself biased or unscientific, as such discussions are part of the broader literature on sexual offending (Kalra & Bhugra, 2013; Scarpati & Pina, 2017).

In addition, it is important to keep in mind that the potential bias in our conviction data is most likely very low. This is because the Nordic countries, including Sweden, are top ranked on the Rule of Law Index that includes criminal justice (World Justice Project, n.d.).

In global comparisons between countries, Sweden also scores very low on measures of racism and discrimination, which means that xenophobia is not a large issue in Sweden compared to many other countries (World Population Review, 2026). We also believe that it is not wrong to discuss cultural differences between countries that may affect the status of women. In many countries outside Europe, gender equality is some of the lowest in the world, whereas it is some of the highest in Sweden.

Finally, there is also an ethical issue in the opposite direction. Stigmatization is a legitimate concern, and researchers must avoid careless generalizations. However, it is also problematic in an ethical sense if robust empirical patterns in serious sexual violence are downplayed only because they are socially uncomfortable. Although the truth sometimes could be inconvenient, it would be unethical not to report it as more knowledge is needed to develop efficient measures in the prevention of rape. The ethical responsibility of the researchers, as well as the editors for criminological journals, is not to avoid difficult findings, but to report them precisely, discuss their limitations clearly, and allow further research to examine the underlying mechanisms.
